# Design-assisted HPLC-UV method for therapeutic drug monitoring of pholcodine, ephedrine, and guaifenesin in biological fluids

**DOI:** 10.1038/s41598-024-78793-6

**Published:** 2024-11-14

**Authors:** Aya Roshdy, Randa Abdel Salam, Ghada Hadad, Fathallah Belal, Heba Elmansi

**Affiliations:** 1 Department of Pharmaceutical Chemistry Faculty of Pharmacy , Horus University, New Damietta, Egypt; 2 Department of Pharmaceutical Chemistry Faculty of Pharmacy, Suez Canal University, Ismailia, Egypt; 3 Department of Pharmaceutical Analytical Chemistry Faculty of Pharmacy, Mansoura University, 35516 Mansoura, Egypt

**Keywords:** Drug interactions, Experimental design, Pholcodine, Ephedrine, Guaifenesin, Biological techniques, Chemistry

## Abstract

Drug-drug interactions may amplify or diminish their intended effects, or even produce entirely new effects. Multicomponent mixture HPLC analysis offers a thorough and effective method for comprehending the makeup and behavior of complicated materials, advancing research and development across a range of scientific and industrial domains. A novel experimental design-assisted HPLC methodology for the concurrent investigation of the drug-drug interaction of pholcodine, ephedrine, and guaifenesin in biological fluids has been established. Rather than the routine methodology, the application of the factorial design-HPLC method offers a powerful and efficient tool for the analysis of these compounds. Both mixed and full factorial designs were employed to assess the impact of variable factors on chromatographic results. Utilizing an isocratic elution mode on a C_18_ column, the chromatographic separation was carried out. 15% Methanol, 5% acetonitrile, and 80% phosphate buffer with 0.1%(v/v) triethylamine set to pH 3 make up the mobile phase flowing at rate 1.0 mL/min. The calibration curves of the drugs show excellent linearity over a concentration ranges: 0.20–13.0 µg/mL for PHO, 0.50–20.0 µg/mL for EPH and 0.70–20.0 µg/mL for GUA with LOQ values of 0.18, 0.38 and 0.50. The fast separation and quantitation in less than 6 min is an advantage. Also, the method includes a robust sample preparation protocol for the analysis of complex biological samples, ensuring high selectivity and precision. The ease, speed and cost-effectiveness of the method are ideal for supporting in vitro studies, including drug-drug interaction investigations, especially in bioanalytical labs.

**Supplementary Information:**

The online version contains supplementary material available at 10.1038/s41598-024-78793-6.

## Introduction

In the fields of pharmacology and medicine, studying drug interactions is an essential issue. Drug-drug interactions occurs when two or more drugs, are administered simultaneously, and their combined effects are different from what would be expected if each one were taken individually. It can affect the safety and efficacy of medications. Some interactions can lead to adverse effects, reduced therapeutic benefits, or even treatment failure^1^. By studying these interactions, healthcare professionals can make more informed decisions about which drugs to prescribe, thereby enhancing patient safety and treatment outcomes^2^. Drug interactions can result from changes in drug absorption, distribution, metabolism, or excretion and may lead to altered drug efficacy or increased risk of side effects^2^. These interactions can lead to various outcomes, including.

- inhibition: one drug may reduce the effectiveness or alter the metabolism of another, potentially reducing the desired therapeutic effects.
- Additive effects: the combined effects of the drugs are simply the sum of their individual effects.
- Synergistic effects: when their combined effects are greater than the sum of their individual effects, leading to an enhanced response, which can be either beneficial or harmful^3^.

The synergistic effects of mixing several active components can result in improved therapeutic advantages, fewer side effects, and better patient compliance. The combination of pholcodine, ephedrine, and guaifenesin is one of such combinations that had caught the interest of analytical researchers^2,4–7^. The analysis of drug combinations is a critical component of pharmaceutical research that helps to enhance patient outcomes and therapeutic efficacy.

The studied combination of pholcodine, ephedrine, and guaifenesin is intended to provide a complete approach to the treatment of respiratory disorders that include cough, congestion, and excessive mucus production^2,4–7^. It was reported that, there was a synergistic enhancement of the drug’s overall therapeutic effects as the cough reflex is suppressed with pholcodine, decreasing the desire to cough. Guaifenesin facilitates mucus thinning and loosening, facilitating easier ejection. Breathing becomes easier when ephedrine clears the nasal passages and opens them up^2,4–7^. Therapeutic Drug Monitoring (TDM) helps maintain drug concentrations within the desired range, enhancing treatment efficacy and reducing the risk of treatment failure or drug-related complications.

**Fig. 1:**
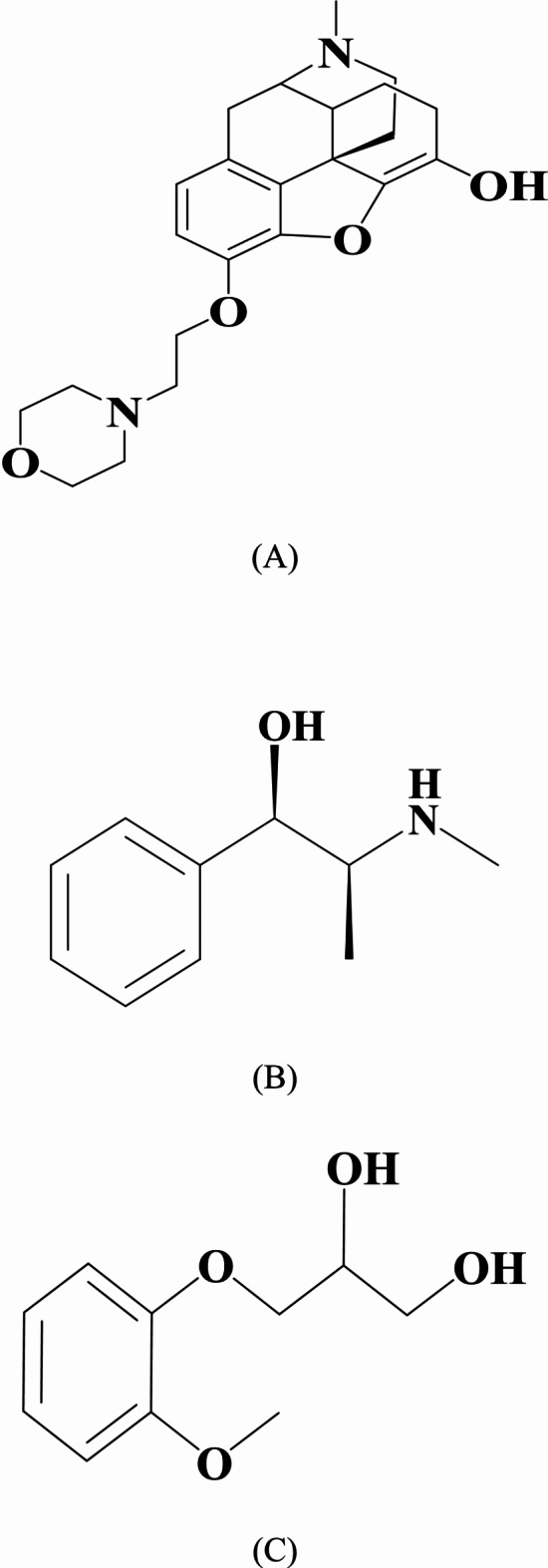
The structural formulae of the studied drugs. (A) pholcodine (B) ephedrine (C) guaifenesin.

Pholcodine (PHL), ((4R,4aR,7 S,7aR,12bS)-3-methyl-9-(2-morpholin-4-ylethoxy)-2,4,4a,7,7a,13-hexahydro-1 H-4,12-methanobenzofuro[3,2-e]isoquinolin-7-ol; hydrate^8^, with a chemical structure illustrated in Fig. 1a. It is a synthetic opioid antitussive medication that is often used to alleviate coughing. It suppresses the cough reflex in the brain without generating considerable central nervous system (CNS) depression^4^. Several methods, including spectrofluorimetric^5,9^and chromatographic^10,11^ were reported for its determination in human plasma.

Ephedrine (EPH) is ((1R,2 S)-2-(methylamino)-1-phenylpropan-1-ol^12^ as shown in Fig. 1b.It is a sympathomimetic amine that functions primarily as a bronchodilator and nasal decongestant. It causes bronchial smooth muscles relaxation and enhanced airflow to the lungs. Furthermore, its vasoconstrictive effects aid in the relief of nasal congestion, making it an important component of cold and allergy treatments^4^. For its determination in plasma, a variety of methods were reported, including, spectrofluorimetry^5^and chromatography^13–16^.

Guaifenesin (GUA) is 3-(2-methoxyphenoxy) propane-1,2-diol^17^, illustrated in Fig. 1c. It is an expectorant that works by decreasing the viscosity of respiratory secretions. It acts through increasing the amount and moisture of respiratory tract mucus, allowing it to be removed more easily through coughing^4^. Regarding its determination in plasma, it was performed by different techniques including spectrofluorimetry^9,18^and chromatography^19–23^.

There have only been a few documented analytical methods for determining such combination. These include spectroscopic^5,7^and HPLC^6,24^ methods.

Experimental design is a crucial strategy in HPLC due to its ability to systematically investigate the effects of multiple factors on chromatographic performance. The use of experimental design (DE) in HPLC optimization provides a systematic and effective approach for obtaining accurate and reproducible results. The method has several merits, including time and resource saving, higher separation efficiency, increased robustness, and data-driven decision-making, all of which contribute to improved analytical accuracy and precision in HPLC applications^25^.

In order to analyze the three drugs simultaneously, a novel experimental design-assisted HPLC method was developed, which benefits because it’s more sensitive, environmentally friendly, having larger working range than reported methods^26^.

## Experimental section

### Apparatus

- The instrument used for the study consisted of a Knauer Chromatograph from Berkin, Germany, fitted with a Knauer D-14,163 injector valve with a 20 µL loop. The eluent was subjected to filtration using 0.45 μm membrane filters manufactured by (Millipore, Cork, Ireland).
- Lab solutions software was used to modify all of the chromatographic data that were collected.
- The factorial design and statistical analysis were performed using Minitab^®^ (Version 16 for Windows, State College, Pennsylvania) statistics software.
- Consort NV P-901 pH Meter (Belgium) was used to adjust pH.
- Centrifuge (2-16P, Germany) and a vortex mixer (IVM-300p, Taiwan) were utilized.

### Materials and reagents

- Pholcodine (PHO) was generously provided by Mash Premiere (El- Obour City, Egypt), its purity was 99.3%.
- Ephedrine (EPH) was kindly supplied by Novartis Pharma AG., Basle, Switzerland), its purity was 99.2%.
- Guaifenesin (GUA) was gratefully given by Novartis Pharma AG., Basle, Switzerland), its purity was 99.2%.
- Solvents (HPLC grade) were purchased from Sigma-Aldrich distribution locations in Cairo, Egypt.
- Coughpent^®^ Syrup; batch No. 2,275,009; purchased from the local market; labeled as having 0.988 mg GUA and 6.55 mg PHO per 5 mL syrup; produced by Global Napi Pharmaceuticals for Penta Pharma, Cairo, Egypt.
- Tusskan^®^ Syrup; batch No. 21,269/2020, manufactured by Hikma Pharmaceutical Company, 6th of October City, Egypt, obtained from the local market, and labelled to contain 7.5 mg EPH and 50 mg GUA per 5 mL syrup.
- Orthophosphoric acid (85%, w/v) was purchased from the distributer of Riedel de Häen (Seelze, Germany).
- ADWIC Co. (Cairo, Egypt) provided sodium hydroxide and sodium dihydrogen phosphate.
- Triethylamine (≥ 99.5%) and Heptane sulfonic acid (Sodium salt) were purchased from the distributer of Sigma-Aldrich (Germany).
- Mansoura University Hospitals, Mansoura, Egypt kindly provided human plasma samples. They were maintained frozen at − 20 °C until used after gentle thawing.

### Standard solutions and mobile phase

#### Standard solutions

To prepare stock solutions, weigh 0.01 gm of each of PHO, EPH, and GUA, then dissolve separately in methanol in 100-mL volumetric flasks to get final concentration of 100.0 µg/mL. The stock solutions kept in refrigerator for two weeks without alteration.

#### Mobile phase

The mobile phase for dosage forms is made up of 15% methanol, 5% acetonitrile, and 80% phosphate buffer with 0.1 (v/v) triethylamine adjusted to pH 3.

While for determination of the drugs in spiked human plasma, the mobile phase is made up of 15% methanol, 5% acetonitrile, and 80% phosphate buffer with 0.8 mL of heptane sulphonic acid (0.025 M) adjusted to pH 3. The solution was ultra-sonicated before filtration via 0.45-µm membrane filters.

### Procedures

#### Construction of calibration graph

Standard solutions of each of PHO, EPH, and GUA were serially diluted to the final concentrations of 0.20–13.0 µg/mL for PHO, 0.50–20.0 µg/mL for EPH, and 0.70–20.0 µg/mL for GUA. Into a series of 10-mL volumetric flasks, serial dilutions were made, and the solutions were made up to the mark with the mobile phase. Under ideal chromatographic conditions, the investigated analytes were injected in triplicate, at a flow rate of 1.0 mL/min, using 20µL aliquots. The average peak area (y) was graphed versus the final drug concentration (C) in µg/mL to get the calibration curves for each analyte. The related regression equation for each drug was then determined.

#### Analysis of synthetic mixtures and laboratory prepared syrup

Aliquots of PHO, EPH, and GUA with different ratios were analyzed in accordance with the **“Construction of calibration graphs”** Section. Guaifenesin and PHO were determined in their combined formulation Coughpent^®^ syrup over the pharmaceutical ratio of (1.0:6.55, respectively) while EPH and GUA were determined in their combined Tusskan^®^ syrup over the pharmaceutical ratio of (1:6.6, respectively). Different aliquots of each preparation was transferred into a 10-mL volumetric flask; diluted to the mark with methanol then sonicated for 10 min. The regression equations were used to compute the nominal content of each preparation.

#### Analysis of PHO, EPH and GUA in human plasma samples

The three analytes were investigated in spiked human plasma samples. Separately, 100 µL aliquots of plasma were transferred into a set of 15 mL centrifuge tubes. To get the final concentrations of 0.10-1.0, 0.20-1.0 and 0.50–1.50 µg/mL for PHO, EPH, and GUA respectively, aliquots from the stock solutions of the three drugs were added, then vortex mixed for 2 min. To separate the drugs from the plasma contents, 4 mL aliquots of methanol were added, vortex-mixed again for 2 min then centrifuged for 30 min at 4000 rpm. Through 0.45 m syringe filters, the clear supernatant layers were filtered. 1.0 mL aliquots from the filtrate were quantitatively added to 10 mL volumetric flasks, and the volume was further completed to the mark with specific mobile phase for biological samples. The drug concentration was measured using the methodology outlined under " **Construction of calibration graphs”** along with a blank sample that was carried out in parallel. Plots of peak area were made against each drug’s concentration.

## Results and discussion

Factorial design permits the simultaneous variation of multiple parameters, including gradient conditions, temperature, flow rate, and composition of the mobile phase. This provides reaching the best conditions for separation efficiency, resolution, and sensitivity. By lowering the number of experiments required, this strategy not only improves the efficiency of method development but also offers a deeper understanding of the relationships between variables. As a result, factorial design makes it easier to validate methods thoroughly, which guarantees accuracy and consistency in analytical applications.

### Preliminary optimization of the experimental parameters

**Fig. 2:**
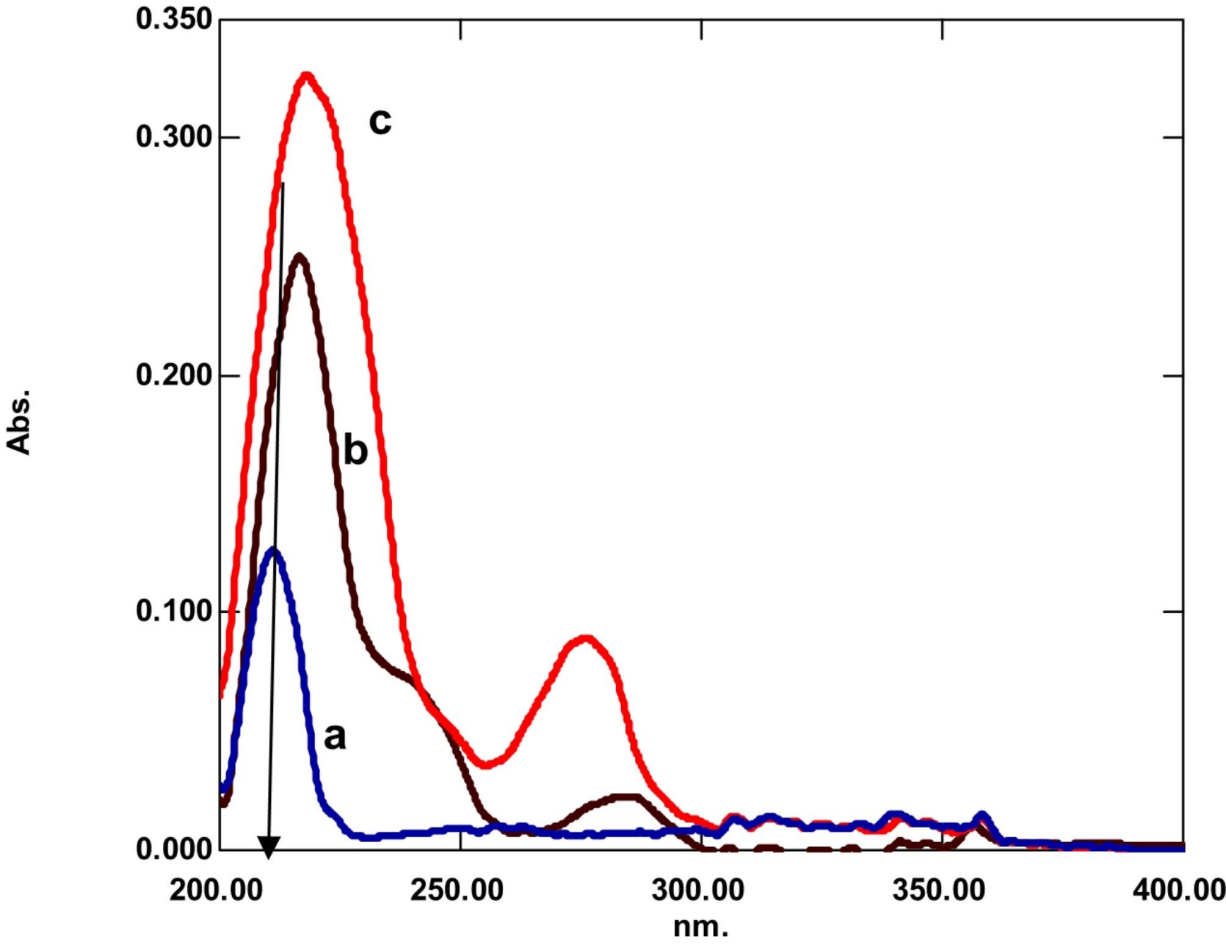
Absorption spectra of the three drugs where (a) is EPH, (b) PHO and (c) is GUA (5 µg/mL each).

- **Detection Wavelength**:
- The UV spectra of three drugs were used to determine the best UV detection wavelength, which was determined to be 210 nm because the three drugs exhibited highest absorbance values at this wavelength (Fig. 2).

- **Mobile Phase Composition**,** pH**,** and Flow rate**:

The mobile phase is a critical component of HPLC method, and its optimization directly influences the efficiency and selectivity of the separation process.

According to previous trials, it was found that decreasing the buffer ratio caused a poor separation of EPH and GUA, hence we used 80 to 85 (V/V) percent buffer.

Experimental design plays a crucial role in HPLC optimization, offering numerous advantages that aid in obtaining accurate and reliable results. By employing a well-structured experimental design, it could enhance the efficiency and effectiveness of the HPLC optimization process, leading to better separations and improved analytical performance^26^.

**Fig. 3:**
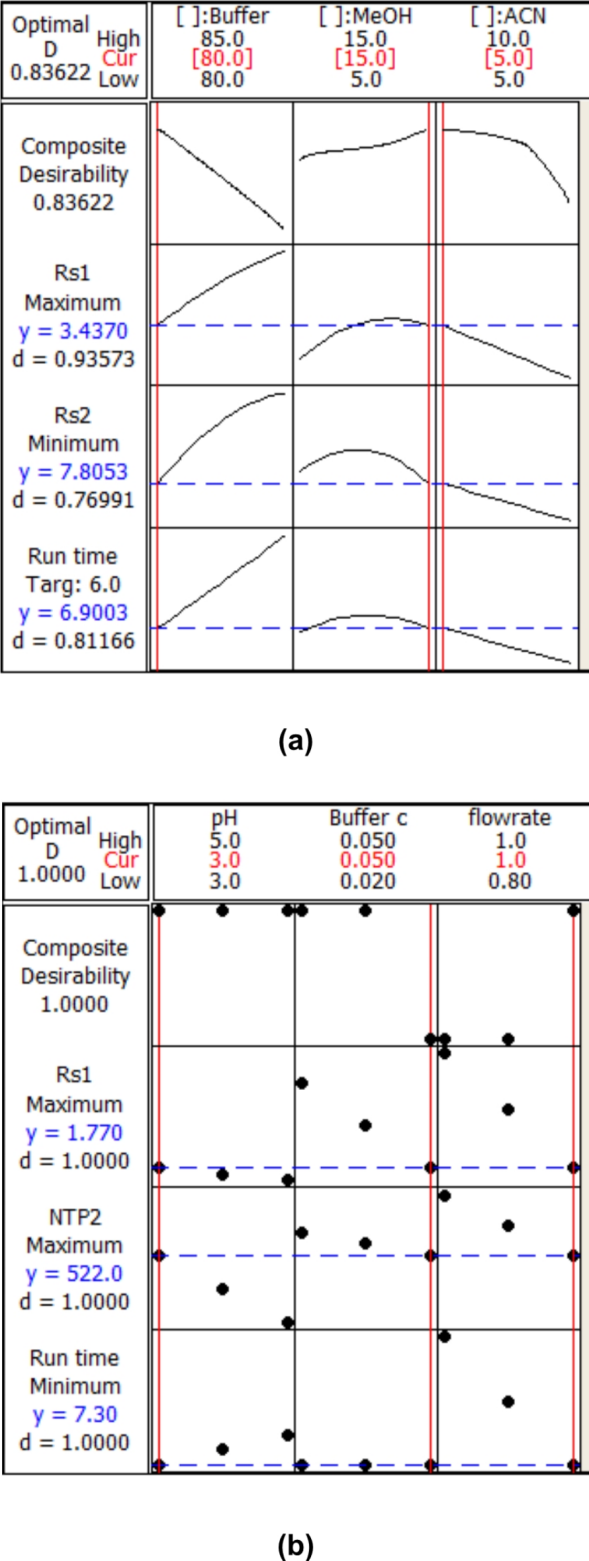
**a** Optimization plot showing the optimum ratio of the mobile phase using the mixture design. **b** 2^3^ FFD Optimization plot showing optimum chromatographic conditions using full factorial design.

Mixture design is a valuable tool for HPLC method optimization, particularly when dealing with complex mixtures. By systematically exploring the factor space and understanding the interactions between components, which can develop robust and efficient HPLC methods yielding accurate and reliable results. It was used to optimize the percent of organic and aqueous solvents in the mobile phase. Buffer ratio was 80–85% (V/V), methanol 5–15% and acetonitrile 5–10% and it was found that the optimum composition of mobile phase was 80% buffer,15% methanol and 5% acetonitrile Fig. 3a.

Additionally, a full factorial design with two levels and four independent variables was utilized to assess and improve the other chromatographic settings, including buffer pH, buffer concentration, and flow rate, which were the most significant factors impacting the HPLC process.

The pH range of potassium dihydrogen phosphate used for the investigation was 3 to 6 because it is the most frequently used pH range. The buffer concentrations were varied from 0.02 to0.05 M and flow rates varying from 0.8 to 1.2 mL/min. Thus, the three independent parameters were buffer pH (A), buffer concentration (B), and flow rate (C). The two levels were thus (-1) for the lower level and (+ 1) for the higher level.

The effect of each level on the responses were investigated using a 2^3^ full factorial design with an 8-run. The independent and dependent variables are shown at two different levels in Table 1.

**Table 1:** 2^3^ dependent responses of experimental factorial designs for the proposed HPLC method.

	Parameters						Optimum solution: pH=3.0, buffer conc=0.05M, Flow rate=1	
							Composite desirability (D)=1.00	
Response	Goal	lower	Target	Upper	Weight	Importance	Predicted responses	*Desirability (d)*
Rs1	Maximize	1.18	1.8	/	1	1	1.7	*1.00*
NTP2	Maximize	217	500	/	1	1	522	*1.00*
Run time	Minimize	/	8	9.5	1	1	7.3	*1.00*

pH: Aqueous mobile phase pH (low level 3 and high level 5).

buffer conc: Aqueous mobile phase buffer concentration (low level 0.02 and high level 0.05).

Flow rate (low level 0.8 and high level 1.0).

Rs1: resolution between PHO & EPH.

NTP2: number of theoretical plates for ephedrine.

An estimated Fisher Statistical Test for Variance Analysis (ANOVA) model^27^ was applied to the responses to ascertain the significance of each factor alone and to monitor the effects of these factors on the responses in addition to their interactions.

The following is the equation for the four-factor experimental design:

R = α0 + α1A + α2B + α3C + α2AB + α2AC + α2BC+ + α2A2 + α2B2 + α2C2.

where R stands for response, α is the regression coefficients, and A, B, and C are buffer pH, buffer concentration and flow rate, respectively.

Achieving the ideal conditions is an important issue. The Minitab response optimizer calculates the composite desirability (D) of the responses, which ranges from zero to one, if the responses are within acceptable limits. Its value should be one or very close to one because one indicates that the state reached is optimum. A zero is inappropriate because multiple responses fall outside of the allowed range.

The optimization plot shows the three factors and their interactions that impact the composite desirability and response to get the best results (Fig. 3-b). The best results were frequently obtained while using the full factorial design.

Half-normal plots and Pareto charts, which are similarly produced by factorial design, reveal that buffer pH (B) and flow rate (C) have statistically significant effects for a 95% confidence level on the NTP of EPH and run time, respectively (Figs. S1 and S2). Which were corroborated by the interaction plots (Fig. S3) demonstrate that raising flow rate and utilizing buffer with pH 3 can reduce run time while maximizing NTP of EPH.

**Fig. 4:**
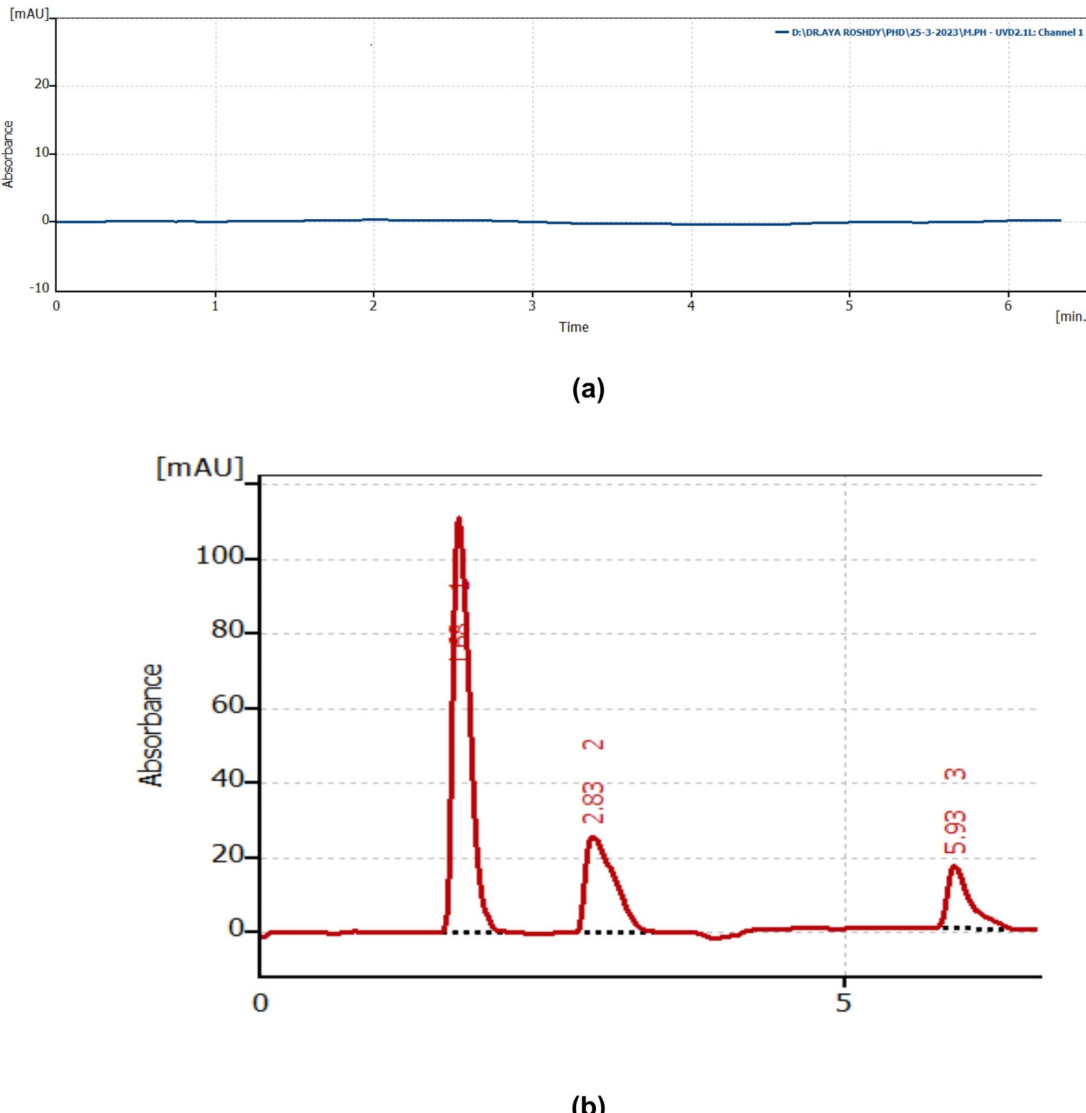
**a** mobile phase. **b** Typical chromatogram under the specified chromatographic conditions (1): PHO (10.0 µg/mL). (2): EPH (10.0 µg/mL) (3): GUA (10.0 µg/mL).

In conclusion, a combination of 15% methanol, 5% acetonitrile, and 80% phosphate buffer with 0.1% v/v triethylamine and pH 3 adjusted in accordance with DOE was used to produce the mobile phase (Fig. 4). According to these conditions, system suitability parameters were abridged in Table 2..

**Table 2.** Parameters of system suitability of the proposed HPLC method

**Parameter**	**PHO**	**EPH**	**GUA**
No of theoretical plates, N	467	522	1537
Capacity factor, k`	2.07	2.14	5.6
Selectivity factor, α	1.04		2.61
Resolution, R_s_	2.8		7.0
Retention time (t_R_), min.	1.68	2.83	5.93
Tailing factor (T)	1.57	2.12	2.18

### Validation parameters

As per the International Conference on Harmonization (ICH) Q2R1 recommendations, validation parameters have been carried out^28^. Regression equations have been derived for investigating into the linearity and ranges for the two drugs after assessing six concentrations for each of PHO, EPH, and GUA. As shown in (Table 3), pure samples of PHO, EPH, and GUA were subjected to the suggested HPLC method, with results covering the ranges of (0.2–13.0 µg/mL, 0.5–20.0 µg/mL, and 0.7–20.0 µg/mL, respectively). The peak area (y) and concentration (c) for the three analytes were plotted, and the correlation coefficients (r) were calculated. The data’s regression equations were calculated and indicated as:

**Table 3:** Analytical performance data using the suggested HPLC method to determine PHO, EPH, and GUA.

Parameter		PHO	EPH		GUA
Linearity range (µg/mL)		0.20-13.0	0.50-20.0		0.70-20.0
Intercept (a)		26.16	24.62		-0.32
Slope (b)		119.72	40.88		24.76
Correlation coefficient (r)		0.9999	0.9999		0.9999
S.D. of residuals (Sy/x)		3.31	2.16		1.92
S.D. of intercept (Sa)		2.23	1.56		1.24
S.D. of slope (Sb)		0.29	0.13		0.11
Percentage relative standard deviation, % RSD		0.45	0.84		0.63
Percentage relative error, % Error		0.18	0.37		0.25
Limit of detection, LOD (µg/mL)		0.06	0.12		0.16
Limit of quantitation, LOQ (µg/mL)	0.18			0.38	0.5

y = 26.16 + 119.72 C (*r* = 0.9999) for PHO.

y = 24.62 + 40.88 C (*r* = 0.9999) for EPH.

y = −0.32 + 24.76 C (*r* = 0.9999) for GUA.

The subsequent mathematical equations^28^ were used to compute the detection limits (DL) and quantitation limits (QL), and the resulting data are provided in (Table 3):

QL = 10 Sa /b DL = 3.3 Sa /b.

A statistical comparison of the obtained results with those resulted from the comparison methods^7,29^, as shown in (Table 4), revealed no statistically significant differences in terms of accuracy and precision^30^. The comparison method for PHO and EPH was a first derivative spectrophotometric method^7^and that for GUA was a native spectrophotometric method that measured the drug at 224.6 nm^29^.

**Table 4:** Assay results for the determination of the studied drugs using the suggested and comparison methods.

Compound	Proposed Method			Comparison methods (5, 18)
	Amount taken (µg/mL)	Amount found (µg/mL)	% Found	% Found
PHO	0.2	0.19	99.55	101.82
	1.0	0.99	99.26	98.18
	7.0	7.01	100.21	100.61
	5.0	5.02	100.37	
	10.0	9.95	99.55	
	13.0	13.02	100.16	
Mean	99.85			100.20
± S.D.	0.45			1.85
t-test	0.31 (2.36)*			
F-test	16.9 (19.3)*			
EPH	0.5	0.49	99.66	98.78
	2.0	1.97	98.30	101.24
	10.0	10.04	100.38	99.59
	15.0	15.05	100.35	
	20.0	19.94	99.72	
Mean	99.68			99.87
± S.D.	0.84			1.25
t-test	0.23(2.45)*			
F-test	2.21 (19.25)*			
GUA	0.7	0.69	99.46	101.54
	1.5	1.51	100.79	98.43
	5.0	5.02	100.34	100.51
	15.0	14.87	99.14	
	10.0	10.02	100.25	
	20.0	20.08	100.40	
Mean	100.06			100.16
± S.D.	0.63			1.58
t-test	0.1 (2.36)*			
F-test	6.29 (19.3)*			

***N.B*** Each results represents the average of three distinct analyses.

*The tabulated t and F values at *P*= 0.05 are the figures included in parenthesis^30^.

Three different concentrations of each drug were analyzed over a period of three consecutive days, ranging from one to three days, in order to confirm the intraday and interday precisions as shown in Table S1.

To test the robustness of the proposed approach; we made minor changes in the experimental conditions. The chromatographic conditions were varied such as buffer percentage (80 ± 1%), pH (3 ± 0.2), and buffer concentration (0.02 ± 0.005 M). The robustness of the approach was demonstrated by finding that such small changes had minimal impact on drug resolution.

## Bioanalytical validation

The bioanalytical validation of the suggested method was performed according to the US FDA guidelines for bioanalytical methods^31^.The following validation characteristics were investigated.

### Selectivity

To make sure that no interferants peaks from endogenous components of plasma were present close to the retention times of the drugs, it was verified using various sources of blank human plasma (Fig. 3a).

### Linearity and range

In spiked plasma samples, high correlation coefficients with linear relationships were obtained using the stated approach. Six calibration standards were analyzed on five different days in order to determine the linearity of each drug. PHO, EPH, and GUA concentrations ranged from 0.20 to 12.5, 0.50–20.0, and 0.70–20.0 µg/mL, respectively. The acceptance criteria for back-calculated standard concentrations were ± 15% deviation from the nominal value except at the LLOQ level; it was set at ± 20%. The regression equations that represent linear relationships are as following:

P_a_ = 16.90 C + 88.57 (*r* = 0.9980) for PHO.

P_a_ = 49.58 C + 6.49 (*r* = 0.9993) for EPH.

P_a_ = 90.06 C – 7.50 (*r* = 0.9995) for GUA.

Where, P_a_ is the peak area, C refers to the drugs concentration (µg/mL), and r represents the correlation coefficients.

### Accuracy and precision

The three analytes: PHO, EPH, and GUA at four distinct concentration levels were estimated for intra- and inter-day precisions and accuracy, showing the QC samples (LLOQ, LQC, MQC, and HQC) in six replicates (*n* = 6) for intra-day and eighteen replicates (*n* = 18) for inter-day. Expressed as percentage recovery and percentage relative standard deviation (% RSD), respectively, the values of accuracy and precision were measured and accepted at ± 15% departure from the nominal values, with the exception of the LLOQ, where it was set at ± 20% (Table S2).

### Stability

The stability of the drugs in human plasma was tested by analyzing them at high and low concentration levels under varied handling and storage settings. Analyte stability was evaluated during handling for 24 h at 4 °C in the autosampler and 24 h at room temperature (benchtop stability). In addition, the stability during storage was assessed at 80 ± 10ºC for 30 days. Every stability assay was tested using three duplicates (Table S3).

### Robustness

It is a measure for the method’ vulnerability to minor deliberate changes that might occur during routine analysis like the effect of buffer (80 ± 1%), pH (3 ± 0.2), and buffer concentration (0.02 ± 0.005 M), after analysis, these parameters were shown to have little or no effect on the percentage of recoveries and RSD values.

### Matrix effect

Analyte peak areas from six different batches at three different concentration levels (LQC, MQC, and HQC) were compared to analyte peak areas from neat samples at the same concentrations, the matrix effect was evaluated. The matrix factor (% CV) for every analyte was determined and found to be less than 4.11%, indicating an insignificant matrix influence (Table S4).

### Dilution integrity

Using spiked human plasma samples at 12.5 µg/mL for PHO and 20.0 µg/mL for each of GUA and EPH, dilution integrity was investigated. Six analyses were made for each of the two and fourfold dilutions of the samples. For each dilution factor, there should be five determinations with an accuracy and precision of 85–115% and ± 15, respectively. (Table S5).

## Applications

### Application to prepared dosage form and synthetic mixtures

Varied ratios of the three examined drugs concentrations were prepared in synthesized mixtures. Examination of these mixes demonstrates the viability of the designed approach for their specific identification. The results in Table S6 showed acceptable standard deviations and good percentage recoveries. Additionally, the comparison of the results with those that were previously reported yields reliable statistical data for the Student’s t-test and Variance ratio F-test^30^.

The suggested approach to analyze the examined drugs in their formulated syrups was successful. According to reports, PHO, EPH and GUA are made into syrups either alone or in combination with other medications. The results obtained and those given utilizing comparison methods^7,29^didn’t show statistical difference (Table S7). The findings were statistically assessed using the Student’s t-test and the Variance Ratio F-test, which showed that the accuracy and precision of the two procedures were comparable^30^.

### Application to spiked human plasma

**Fig. 5:**
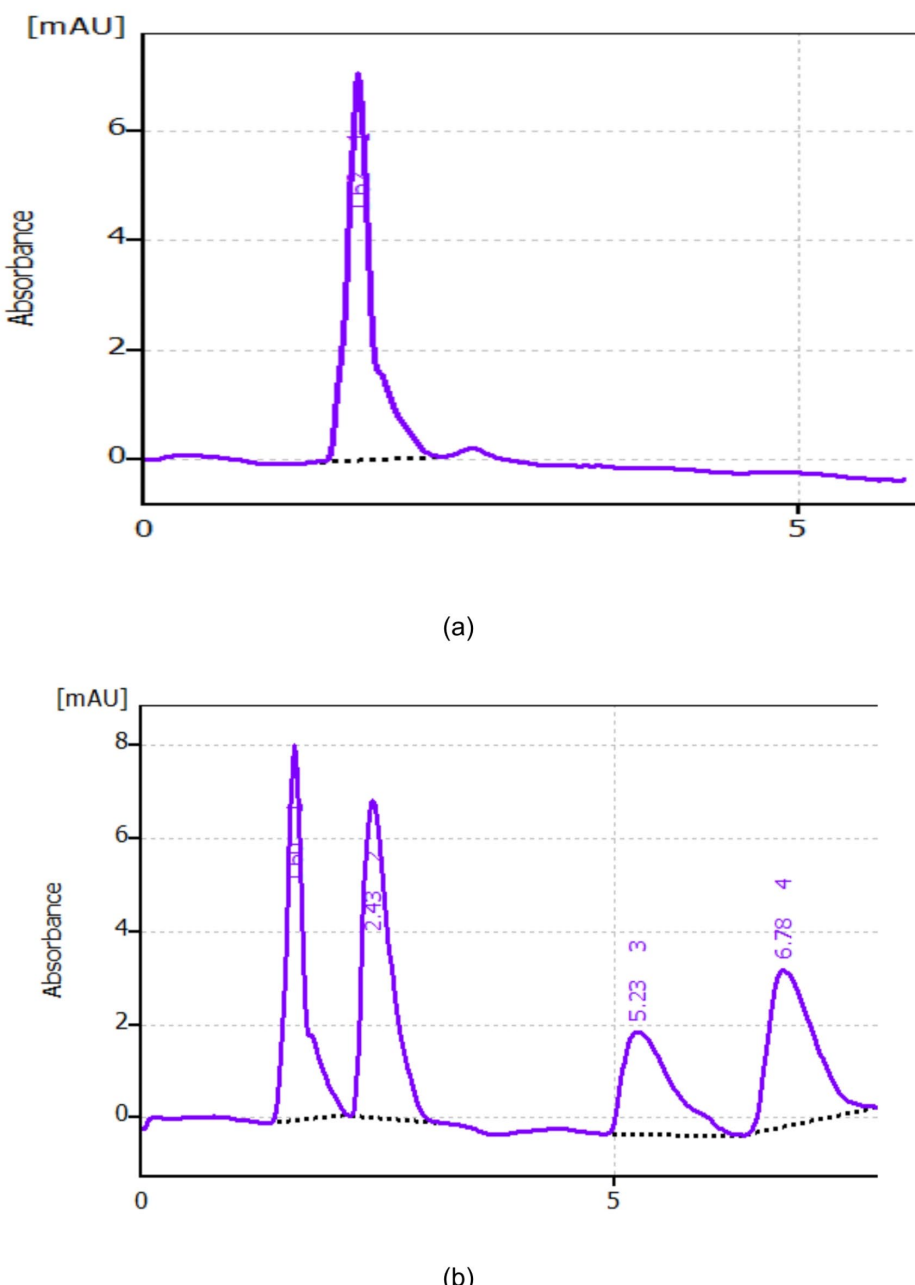
**a** Blank plasma. **b** Typical drug chromatogram in spiked human plasma at the specified chromatographic conditions: (1): plasma peak (2): PHO (0.5 µg/mL). (3): EPH(1.0 µg/mL) (4): GUA (1.0 µg/mL).

It was reported that the maximum plasma concentration (C_max_) of PHO was 12.8 µg/mL after on single dose (5). Adults’ GUA (C_max_) concentration after 400 mg of the drug was 2.316 µg/mL^32^. The suggested method’s sensitivity for PHO, EPH and GUA was down to 0.1 µg/mL, 0.2 µg/mL and 0.5 µg/mL, respectively. Therefore, estimating the biological levels of the three drugs was acceptable by the current HPLC approach. By graphing the peak area with the drug concentration, a linear relationship has been established in plasma samples spiked with PHO, EPH and GUA using the proposed methodology. Table 5. shows the good percentage recovery for different samples spiked with the three analytes. A chromatogram showing well resolved peaks in spiked human plasma is illustrated in Fig. 5.

**Table 5.** Assay results for the indicated drugs in human plasma sample using the suggested method

**Spiked human plasma**	**Amount. taken (** **mg/mL)**			**% Found**		
	**PHO**	**EPH**	**GUA**	**PHO**	**EPH**	**GUA**
	0.10	0.20	0.50	99.20	98.35	101.22
	0.30	0.50	1.00	98.80	101.62	98.01
	0.50	0.70	0.70	101.30	99.34	100.70
	1.00	1.0	1.50	99.79	99.98	100.60
**Mean %**				99.77	99.82	100.13
**± S.D.**				1.10	1.37	1.44
**%RSD**				1.10	1.37	1.44
**%Error**				0.55	0.69	0.72

Linear regression analysis of the data gave the following equation:

Y = 45.79 + 92.79 C (*r* = 0.9990) For PHO GUA.

Y = 10.30 + 42.70 C (*r* = 0.9991) For EPH.

Y = -20.3 + 83.75 C (*r* = 0.9995) For GUA.

## Conclusion

The implementation of an experimental design-HPLC approach for determining pholcodine, ephedrine, and guaifenesin in biological fluids has shown significant advantages. The chromatographic separation was carried out. 15% Methanol, 5% acetonitrile, and 80% phosphate buffer with 0.1%(v/v) triethylamine set to pH 3. The developed method allowed their separation and quantitation in less than 6 min. The experimental design methodology enabled a systematic evaluation of crucial technique parameters, resulting in the optimization and improvement of the HPLC method’s separation efficiency and sensitivity. The HPLC system proved to be appropriate for simultaneous analysis of the target drugs. Over a specific concentration range, the approach demonstrated excellent linearity and sensitivity, allowing accurate measurement of analytes in varied sample matrices. According to statistical data analysis, the approach is repeatable, precise, accurate, and robust, enables the pharmaceutical industry to employ therapeutic drug monitoring for in-patients.

## Electronic supplementary material

Below is the link to the electronic supplementary material.

Supplementary Material 1

## Data Availability

Data availabilityThe datasets generated and/or analyzed during the current study are available from the corresponding authoron reasonable request.
