# Supplementary material for: Design-assisted HPLC-UV method for therapeutic drug monitoring of pholcodine, ephedrine, and guaifenesin in biological fluids
Source: Sci Rep. 2024 Nov 14;14:27933. doi: 10.1038/s41598-024-78793-6 (PMC11560963; doi:10.1038/s41598-024-78793-6)
Supplement: Supplementary file 1 — Supplementary Material 1 [file 41598_2024_78793_MOESM1_ESM.docx]

**Supplementary File for:**

**Design-Assisted HPLC-UV Method for Therapeutic Drug Monitoring of Pholcodine, Ephedrine, and Guaifenesin in Biological Fluids**

**Supplementary Figures:**

- Figures S1: 2^3^ FFD Plots of half-normality for chromatographic responses according to data means.
- Figure S2: Effects on the chromatographic responses at alpha=0.05 are shown in 2^3^ FFD Pareto plots.
- Figure S3: For chromatographic responses, 2^3^ FFD full interaction graphs per data means type are shown.

**Figure S1**: 2^3^ FFD Plots of half-normality for chromatographic responses according to data means.

**Figure S2:** Effects on the chromatographic responses at alpha=0.05 are shown in 2^3^ FFD Pareto plots.

**Figure S3:** For chromatographic responses, 2^3^ FFD full interaction graphs per data means type are shown.

**Supplementary Tables:**

**Table S1: Precision data to determine the studied compounds using the suggested method.**

| Drug | Conc.  (μg/mL) | Intra-day | | | Inter-day | | |
| --- | --- | --- | --- | --- | --- | --- | --- |
|  |  | Mean ± S.D | %RSD | % error | Mean ± S.D | %RSD | % error |
| PHO | 3.0 | 99.35 ± 0.56 | 0.56 | 0.33 | 99.68 ± 0.64 | 0.64 | 0.37 |
|  | 5.0 | 99.81± 0.30 | 0.31 | 0.18 | 100.32 ± 0.83 | 0.83 | 0.48 |
|  | 10.0 | 99.62 ± 0.47 | 0.47 | 0.27 | 100.55 ± 0.60 | 0.61 | 0.35 |
| EPH | 5.0 | 99.81± 0.27 | 0.27 | 0.16 | 99.39 ± 0.62 | 0.62 | 0.36 |
|  | 10.0 | 99.45 ± 0.27 | 0.27 | 0.16 | 99.98± 0.42 | 0.42 | 0.24 |
|  | 15.0 | 99.81 ± 0.30 | 0.30 | 0.17 | 100.6 ± 0.52 | 0.52 | 0.30 |
| GUA | 5.0 | 99.39 ± 0.55 | 0.55 | 0.32 | 100.07 ± 0.92 | 0.92 | 0.53 |
|  | 10.0 | 98.64 ± 0.43 | 0.43 | 0.25 | 100.15 ± 0.63 | 0.62 | 0.36 |
|  |  | 99.54 ± 0.61 | 0.61 | 0.35 | 99.44 ± 1.00 | 1.00 | 0.58 |

***N. B.***  Each result is the average of three separate determinations.

**Table S2: Precision data for the determination of the studied drugs in spiked human plasma by the proposed method.**

| **Analyte** | **Concentration (µg/mL)** | | **Intra-day** | | **Inter-day** | |
| --- | --- | --- | --- | --- | --- | --- |
|  |  |  | **Mean (%)** | **%RSD** | **Mean (%)** | **%RSD** |
| **PHO** | **LLOQ** | 0.20 | 100.10 | 3.63 | 99.89 | 6.02 |
|  | **LQC** | 0.60 | 100.57 | 8.09 | 101.75 | 4.70 |
|  | **MQC** | 10.0 | 100.98 | 6.06 | 99.83 | 4.20 |
|  | **HQC** | 12.5 | 100.85 | 4.11 | 100.69 | 5.60 |
| **EPH** | **LLOQ** | 0.50 | 95.93 | 7.78 | 99.77 | 8.51 |
|  | **LQC** | 1.20 | 98.61 | 4.26 | 100.71 | 5.31 |
|  | **MQC** | 15.0 | 98.92 | 5.74 | 98.8 | 5.70 |
|  | **HQC** | 20.0 | 100.53 | 5.50 | 100.59 | 4.04 |
| **GUA** | **LLOQ** | 0.70 | 99.05 | 7.25 | 99.43 | 6.39 |
|  | **LQC** | 2.10 | 98.15 | 7.08 | 101.7 | 8.83 |
|  | **MQC** | 15.0 | 98.50 | 4.25 | 97.97 | 4.94 |
|  | **HQC** | 20.0 | 99.62 | 3.95 | 98.67 | 5.03 |
| **N** |  | | 6 | | 18 | |

**Table S3: Stability results in human plasma under various conditions.**

| **Analyte** | **Concentration (µg/mL)** | | **Short-term stability at room temperature**  **(24 hr)** | | **Long term stability at -80 ºC (30 days)** | | **Processed sample stability at 4ºC (24 hr)** | |
| --- | --- | --- | --- | --- | --- | --- | --- | --- |
|  |  |  | **Accuracy (%)** | **%RSD** | **Accuracy (%)** | **%RSD** | **Accuracy (%)** | **%RSD** |
| **PHO** | **LQC** | **0.60** | 98.83 | 0.29 | 99.21 | 0.66 | 99.00 | 0.53 |
|  | **HQC** | **12.5** | 102.57 | 3.10 | 103.53 | 4.11 | 101.31 | 2.89 |
| **EPH** | **LQC** | **1.20** | 100.90 | 2.33 | 101.13 | 5.14 | 101.00 | 4.36 |
|  | **HQC** | **20.0** | 99.40 | 4.65 | 100.53 | 5.75 | 100.33 | 3.17 |
| **GUA** | **LQC** | **2.10** | 96.67 | 2.15 | 98.67 | 4.04 | 97.00 | 2.65 |
|  | **HQC** | **20.0** | 97.67 | 2.31 | 98.60 | 3.42 | 98.63 | 2.47 |

**Table S4: The matrix effect influence in spiked human plasma samples at different concentrations of the studied analytes.**

| **Analyte** | **Concentration (µg/mL)** | | **Mean (%)** | **CV (%)** |
| --- | --- | --- | --- | --- |
| **PHO** | **LQC** | **0.60** | 102.05 | 4.0 |
|  | **MQC** | **10.0** | 101.33 | 2.52 |
|  | **HQC** | **12.5** | 99.87 | 1.86 |
| **EPH** | **LQC** | **1.20** | 100.00 | 2.65 |
|  | **MQC** | **15.0** | 101.10 | 2.07 |
|  | **HQC** | **20.0** | 100.67 | 3.22 |
| **GUA** | **LQC** | **2.10** | 96.67 | 2.08 |
|  | **MQC** | **15.0** | 98.67 | 3.51 |
|  | **HQC** | **20.0** | 98.33 | 4.11 |
| **N** |  | | 6 | |

**Table S5: The Dilution integrity results in human plasma at different concentrations**

| **Analytes** | **Concentration (µg/mL)** | **2-fold dilution** | | **4-fold dilution** | |
| --- | --- | --- | --- | --- | --- |
|  |  | **Mean (%)** | **CV (%)** | **Mean (%)** | **CV (%)** |
| **PHO** | **12.5** | 98.96 | 3.12 | 101.4 | 3.50 |
| **EPH** | **20.0** | 97.30 | 3.70 | 97.67 | 4.20 |
| **GUA** | **20.0** | 99.00 | 3.00 | 100.33 | 3.50 |
| **N** |  | 6 | | 6 | |

**Table S6: Assay results for the determination of the studied drugs in laboratory-prepared mixtures by the proposed HPLC method.**

| **% Found** | | | **Amt. taken (μg/mL)** | | |  |
| --- | --- | --- | --- | --- | --- | --- |
| **GUA** | **EPH** | **PHO** | **GUA** | **EPH** | **PHO** | **Synthetic mixture** |
| 101.77 | 98.17 | 101.83 | 5.0 | 1.00 | 5.00 | 1 |
| 98.23 | 100.51 | 98.26 | 10.0 | 10.00 | 7.00 | 2 |
| 100.59 | 99.78 | 100.23 | 15.0 | 15.00 | 13.00 | 3 |
| 100.20 | 99.49 | 100.11 |  | | | **Mean %** |
| 1.80 | 1.20 | 1.79 |  |  |  | **± S.D.** |

**Table S7: Application of the suggested method to Coughpent® and Tusskan® syrup:**

| **Comparison methods**  **[7, 29]** | | | | | | **Proposed method** | | | | | **Compound** | | | |
| --- | --- | --- | --- | --- | --- | --- | --- | --- | --- | --- | --- | --- | --- | --- |
| **% found** | | **Amount found**  **(μg/mL)** | | **Amount taken**  **(μg/mL)** | | **% found** | | **Amount found**  **(μg/mL)** | | **Amount taken (μg/mL)** | | |  | |
| 98.49 | | 4.94 | | 5.00 | | 98.76 | | 1.293 | | 1.31 | | | **PHO in Coughpent®**  **Syrup** | |
| 101.01 | | 10.101 | | 10.00 | | 101.65 | | 1.67 | | 1.64 | | |  |  |
| 99.70 | | 14.95 | | 15.00 | | 99.21 | | 1.954 | | 1.97 | | |  |  |
| 99.73 | |  | |  | | 99.87 | |  | |  | | | **Mean** | |
| 1.26 | |  | |  | | 1.57 | |  | |  | | | **± S.D.** | |
| 0.14 (2.78) | | | | | | | | | | | | **t** | |  |
| 1.53 (19.0) | | | | | | | | | | | | **F** | |  |
| 98.40 | | 9.84 | | 10.00 | | 101.33 | | 0.202 | | 0.20 | | | **GUA in Coughpent®**  **syrup** | |
| 101.55 | | 30.46 | | 30.00 | | 98.67 | | 0.246 | | 0.25 | | |  |  |
| 99.47 | | 49.73 | | 50.00 | | 100.44 | | 0.301 | | 0.30 | | |  |  |
| 99.81 | |  | |  | | 100.15 | |  | |  | | | **Mean** | |
| 1.6 | |  | |  | | 1.35 | |  | |  | | | **± S.D.** | |
| 0.32(2.78) | | | | | | | | | | | | **t** | |  |
| 1.4(19.00) | | | | | | | | | | | | **F** | |  |
| 98.40 | 9.84 | | 10.00 | | 98.15 | | 6.48 | | 6.6 | | | **GUA in Tusskan® syrup** | |  |
| 101.55 | 30.46 | | 30.00 | | 101.85 | | 13.44 | | 13.2 | | |  |  |  |
| 99.47 | 49.73 | | 50.00 | | 99.38 | | 19.67 | | 19.8 | | |  |  |  |
| 99.81 |  | |  | | 99.79 | |  | |  | | | **Mean** | |  |
| 1.6 |  | |  | | 1.88 | |  | |  | | | **± S.D.** | |  |
| 0.02(2.78) | | | | | | | | | | | | **t** | |  |
| 1.38(19.0) | | | | | | | | | | | | **F** | |  |
| 98.23 | 98.23 | | 100.0 | | 101.22 | | 1.012 | | 1.0 | | | **EPH in Tusskan® syrup** | |  |
| 99.94 | 199.88 | | 200.0 | | 98.78 | | 1.975 | | 2.0 | | |  |  |  |
| 100.13 | 300.39 | | 300.0 | | 100.41 | | 3.012 | | 3.0 | | |  |  |  |
| 99.43 |  | |  | | 100.14 | |  | |  | | | **Mean** | |  |
| 1.05 |  | |  | | 1.24 | |  | |  | | | **± S.D.** | |  |
| 0.85 (2.78) | | | | | | | | | | | | **t** | |  |
| 1.39(19.00) | | | | | | | | | | | | **F** | |  |

***N. B.*** Each result is the average of three separate determinations.

*The figures between parentheses are the tabulated t and F values at P = 0.05 [[30](#_ENREF_30)].
